# Oral Anticoagulants for Newly Detected Atrial Fibrillation in Cryptogenic Stroke Patients: Still a Bitter Pill to Swallow Quickly

**DOI:** 10.19102/icrm.2021.121206

**Published:** 2021-12-15

**Authors:** Rahul N. Doshi, Amy Kleinhans

**Affiliations:** ^1^Cardiac Arrhythmia Group, Cardiovascular Center of Excellence, HonorHealth, Scottsdale, AZ, USA

**Keywords:** Atrial fibrillation, oral anticoagulation, stroke

In this issue of the journal, Kloosterman et al. provide us with some important data to “close the loop” between robust monitoring for the diagnosis of atrial fibrillation (AF), patients who suffer from cryptogenic stroke, and the utilization of oral anticoagulant (OAC) therapy after diagnosis.^1^ In their single-center experience, 30% of patients monitored over a mean of 19 months were diagnosed with previously undetected AF. Almost 90% of these patients were started on OACs, which effectively reduced the likelihood of recurrent stroke relative to that in patients without AF detected. Patients not started on OACs had a much higher rate of recurrent stroke. In this relatively small dataset, there were no significant differences between patients who were started on OACs and those who were not. This of course then begs the question—why not apply this strategy uniformly?

It is well established that patients who suffer a cryptogenic stroke have a high incidence of AF and that more rigorous monitoring leads to increased detection. The Cryptogenic Stroke and Underlying Atrial Fibrillation (CRYSTAL AF) study demonstrated a 12.4% incidence in patients implanted with an implantable loop recorder (ILR) over a 12-month period.^2^ At three years, approximately 30% were diagnosed with AF,^3^ in keeping with the results presented here. It is important to remember that, despite the high incidence of AF detected in this population, two clinical trials of empiric OAC therapy after stroke have failed to show a benefit.^4,5^ The European Society of Cardiology 2020 guidelines for AF monitoring after cryptogenic stroke give a 1B recommendation for short-term (72 hours) monitoring and an IIB indication for long-term monitoring with ambulatory monitors or an ILR.^6^

Of course, much of the uncertainty regarding utilization surrounds which specific populations are appropriate to focus on—which at least in part is related to the overall risk–benefit profile of the therapy (OAC) and not the diagnostic approach (eg, ILR). A lower-risk population may have AF diagnosed, but treating them with an OAC may not result in a benefit, or the risks of OAC therapy may outweigh any potential benefit. The recently reported AF Detected by Continuous Electrocardiographic Monitoring (LOOP) study illustrates this point.^7^ This multicenter Danish study randomized more than 6,000 patients aged 70 to 90 years with at least one risk factor for stroke (eg, hypertension, diabetes, prior stroke, or heart failure) in a 1:3 fashion to ILR implantation versus usual care. OAC therapy was recommended for patients with episodes lasting more than six minutes in duration. The Asymptomatic Atrial Fibrillation and Stroke Evaluation in Pacemaker Patients and the Atrial Fibrillation Reduction Atrial Pacing Trial (ASSERT) has previously demonstrated a 2.5-fold increase in stroke risk for subclinical AF detected on cardiac implantable electronic device interrogation, with a six-minute duration associated with an increased risk.^8^ Despite AF being detected at a similar rate of 31.8% over the mean follow-up of 64.5 months compared to a rate of 12.2% in the control group, with most of these patients started on OACs, there was no significant reduction in the risk of stroke or arterial embolism in the ILR group (4.5% vs. 5.6%; p = 0.11). As expected, there was a 1% increased absolute risk of bleeding that was also not statistically significant. This trial and data from other such trials lead us to two important limitations when applying these types of data to our patients. The first is that, the shorter the duration of AF detected, the smaller the risk. The second is that, the shorter the duration, the more strongly reduced the specificity of the diagnostic tool is. Thus, within these datasets, there are patients (with higher-risk factors, longer durations of arrhythmia) who have a greater risk and drive away the potential benefits of instituting therapy. It is important to point out that, in the LOOP study, the cumulative risk curves for ischemic stroke and cardiovascular death did continue to diverge beyond the mean follow-up, whilst the risk of all-cause death remained identical.^7^

This illustrates the importance of studies such as that presented here by Kloosterman et al.^1^ They demonstrate that the treatment initiated based on a new diagnosis of AF resulted in a tangible benefit, reducing the stroke risk compared to that of the population without AF. Risk factors for stroke and CHA_2_DS_2_-VASc scores were similar across all groups. However, a significant limitation of these data is that, of the six strokes that occurred in patients with AF not on OACs, four occurred prior to the diagnosis of AF. If we remove these patients, that leaves two of 10 patients who were diagnosed with AF prior to stroke who were not on OACs. This 20% incidence is higher than the 5% rate in the overall population, but the numbers are too small to draw any reasonable conclusions. Nevertheless, the authors should be congratulated on a significant contribution to the field, one that should spur larger prospective studies to validate these findings.

The authors do discuss the importance of relative time independence of stroke and AF. Recently, the idea of the timing of AF burden in relation to stroke has again been questioned. In trials such as ASSERT, the temporal relationship of an episode of AF with the occurrence of stroke is unclear, thus raising the question of whether AF is not a causal factor but instead related to the presence of an atrial myopathy that is associated with the risk of stroke. Recently, Singer et al. examined the presence of longer durations of AF (> 5.5 hours on any day) and the association of AF.^9^ These authors demonstrated that the presence of this AF burden is only associated with an increased risk of stroke one to five days prior to the event and not at any other time within 120 days prior. Specifically, the occurrence of more than 5.5 hours of AF one to five days prior to an event was associated with a fivefold increased risk of stroke. Also, AF episodes lasting more than 23 hours at any time were associated with a fivefold risk of stroke. Factors such as these illustrate the complexity of variables likely associated with stroke risk related to AF, thus making it harder to reach a clear consensus.

The use of OACs remains a challenge, even in known AF patients at high risk of stroke. Compliance remains a significant issue despite novel OACs compared to warfarin. Rapid initiation of such therapy after a diagnosis, despite the widespread use of remote monitoring, remains difficult, with multiple barriers, including protocol utilization, staffing requirements, pharmacy barriers and costs, and patient perceptions (often justified) of bleeding risk. Cost remains a significant contributor to poor adherence even despite the multiple drugs currently available and remains associated with the discontinuation of therapy.^10^ The data presented by Kloosterman et al. are a necessary step to support large-scale randomized trials that may lead to more widespread utilization, better patient education, and reductions in patient costs. Finally, non-pharmacologic strategies such as left atrial appendage occlusion should be evaluated prospectively in similar cohorts, rather than just assuming they are equivalent to OAC therapy.

For our patients’ benefit, let us act quickly.
